# BUILDING DISASTER RESILIENCE FOR OLDER ADULTS AGING IN PLACE: THE ROLE OF COMMUNITY-BASED ORGANIZATIONS

**DOI:** 10.1093/geroni/igz038.1234

**Published:** 2019-11-08

**Authors:** Claire Pendergrast, Basia Belza, Ann Bostrom, Nicole Errett

**Affiliations:** University of Washington, Seattle, Washington, United States

## Abstract

Older adults are more susceptible to adverse health outcomes during and after a disaster compared with their younger counterparts. Developing community resilience, or strengthening communities to reduce the negative impacts of disasters, has the potential support older adults’ health and well-being. Community-based organizations (CBOs), such as senior centers and Villages, provide social services and programming that support aging in place and may support older adults’ resilience to disasters. This study examines CBO leadership perspectives on the role of CBOs in building disaster resilience for older adults aging in place, as well as perceived barriers and facilitators to incorporating disaster resilience activities into organizational programming. In-depth interviews were conducted with a purposive sample of staff-members of CBOs serving older adults aging in place in King County, Washington. Participants included representatives from 14 organizations that varied in size, geographic setting, organizational structure, and ethnic, linguistic, and socio-economic backgrounds of organizational members. The sample included five government-run senior centers, seven non-profit senior centers, and two Villages. Interviews were audio-recorded and transcribed verbatim. We used a combined inductive and deductive approach to code and thematically analyze the data. Results indicate that local context, leadership risk perception, collaborations, and existing services and programming influence CBOs’ willingness to engage in activities supporting disaster resilience for older adults aging in place. Findings suggest that CBOs supporting aging in place may support disaster resilience for older adults by serving as a trusted source of disaster preparedness information and tailoring disaster-related messages for an older adult audience.

